# Surgery for Bacterial Endocarditis Complicated by Diffuse Alveolar Hemorrhage

**DOI:** 10.1155/2019/3427381

**Published:** 2019-05-28

**Authors:** Kazuhiro Kurisu, Hiroshi Mitsuo, Yasutaka Ueno

**Affiliations:** Department of Cardiovascular Surgery, Shimonoseki City Hospital, Shimonoseki, Japan

## Abstract

Diffuse alveolar hemorrhage is a very rare but potentially lethal condition resulting from various disorders. We report the case of a patient who suffered diffuse alveolar hemorrhage subsequent to bacterial endocarditis and survived aortic valve replacement, which was applied after improvement in respiratory distress. We believe that the strategy of respiratory functional recovery by aggressive rehabilitation is essential for the achievement of a successful surgical outcome in patients with alveolar hemorrhage.

## 1. Introduction

Diffuse alveolar hemorrhage (DAH) is a very rare but potentially fatal condition [1–3]. Its etiology is wide and includes, *inter alia*, autoimmune vasculitis, infection, drug toxicities, and organ transplantation [1–3]. As this condition is caused by bleeding into the alveoli resulting from the disruption of the alveolar-capillary basement membrane, respiratory distress is frequently a serious concern [1–3]. Cardiac valve surgery should be avoided in this situation because it may lead to further bleeding and exacerbated respiratory failure. We describe a patient who suffered DAH following bacterial endocarditis and survived aortic valve replacement after improvement in respiratory distress syndrome.

## 2. Case Presentation

**Figure 1:**
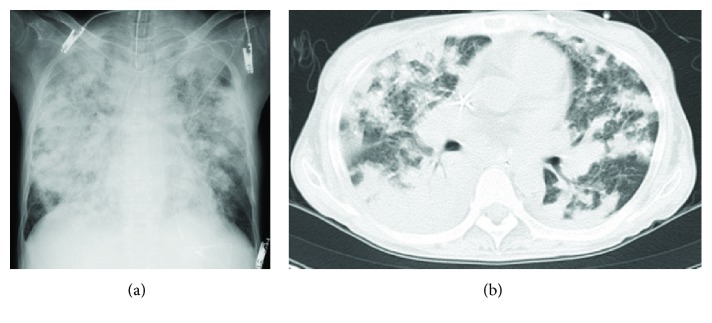
Preoperative chest radiography (a) and computed tomography (b) showing bilateral diffuse consolidation.

A 45-year-old woman, on a regimen of steroids for a diagnosis of Behçet's disease or systemic lupus erythematosus, was admitted with pyrexia, cough, and worsening dyspnea of a week's duration. She had a history of small bowel resection for intestinal necrosis and had been on parenteral nutrition since then. She presented a fever of 39.1°C, tachycardia of 116/min, and hypoxia with oxygen saturation of 94% in nasal oxygen cannula. A grade 3/6 regurgitant diastolic murmur was heard in the left third intercostal space on auscultation. She soon fell into respiratory failure and was immediately supported by mechanical ventilation. The ratio of the arterial oxygen pressure and the fraction of inspired oxygen (PaO_2_/FiO_2_) was initially calculated as 105. Chest radiography and computed tomography revealed diffuse extensive consolidation in bilateral fields corresponding with DAH (Figure 1). This diagnosis was confirmed by bronchoalveolar lavage with increasing bloody secretion in three consecutive aliquots. In a peripheral blood examination, the white blood cell count was 11,540/mm^3^, hemoglobin 6.6 g/dL, and platelet count 132,000/mm^3^. Aspartate aminotransferase, alanine aminotransferase, and lactate dehydrogenase were 18, 16, and 164 IU/L, respectively. Renal function was slightly affected, with a creatinine level of 1.5 mg/dL. C-reactive protein was elevated to 12.3 mg/dL. Blood cultures afterward grew *Staphylococcus warneri*. Echocardiography showed aortic regurgitation and a mobile vegetation of 15 × 9 mm in size on the commissure between the left and right coronary cusps. Left ventricular function was preserved with an ejection fraction of 62%. The patient was diagnosed with bacterial endocarditis [4] complicated with respiratory distress related to the DAH.

At this point, she was judged not to tolerate cardiac surgery because of the exacerbating bleeding and respiratory failure related to heparinized extracorporeal circulation. The management plan was as follows: (1) antibiotic and inotropic therapies with diuretics for the bacterial endocarditis and heart failure, (2) steroid pulse and hemostatic drug therapies for DAH, and (3) postponement of cardiac surgery until sufficient improvement in respiratory function unless circulation collapses. Nevertheless, the respiratory function initially made little improvement despite adequate cardiac condition. Because the clot in the alveolar space could not be excreted in the usual supine position, a tracheotomy followed by aggressive positional changes to include an abdominal position was planned. The tracheotomy was performed on hospital day 12. Thereafter, respiratory function gradually improved although mechanical ventilation was still needed, and the PaO_2_/FiO_2_ ratio reached 356 on hospital day 31. Methylprednisolone was then adjusted to a maintenance dose of 40 mg/day, and daptomycin was administered at 210 mg/day. The white blood cell count was 8,210/mm^3^ with a shift to the left, and C-reactive protein was 10.2 mg/dL. Repeated echocardiography revealed worsening aortic regurgitation suggesting the disruption of the commissure between the left and right coronary cusps and the mildly enlarged vegetation. We decided to perform the valve surgery in this timing when the respiratory distress improved.

J shaped partial sternotomy was performed between the second intercostal space and the xiphoid process. A cardiopulmonary bypass was equipped with right femoral artery perfusion and right atrial drainage. A 19 mm St. Jude Medical mechanical prosthesis (St. Jude Medical, St. Paul, MN, USA) was inserted with subannular reinforcement [5] using a rifampicin-soaked woven Dacron graft (Vascutek, Terumo, Inchinnan, UK) to prevent valve detachment associated with Behçet's disease. Respiratory function was well maintained, and mechanical ventilation support was discontinued on the seventh postoperative day. The control of infection was satisfactory. The patient was discharged home following closure of the tracheotomy. Methylprednisolone therapy was continued at the maintenance dose of 40 mg/day during the operative and postoperative days. Daptomycin was also used at the same dose of 210 mg/day, and ampicillin sodium/sulbactam sodium was added only on the day of operation.

## 3. Discussion

DAH is a potentially fatal condition because the respiratory function is seriously impaired by bleeding into the alveoli resulting from the disruption of the alveolar-capillary basement membrane [1–3]. Its etiology is wide and includes autoimmune vasculitis, infection, drug toxicities, and organ transplantation [1–3]. In this particular case, we speculate the autoimmune vasculitis resulted from inflammation provoked by the bacterial infection and caused the disruption of the alveolar-capillary basement membrane and further alveolar hemorrhage. Systemic lupus erythematosus probably participated in the mechanism of this illness. DAH resulting from bacterial endocarditis is extremely rare, and this combination has been reported only once, by Wu et al. [6], who treated their patient with intravenous antibiotic therapy but not surgically. To our knowledge, the present patient is the first to survive surgery for infective endocarditis complicated with DAH.

The reported in-hospital mortality of DAH ranged from roughly 25% to 50% [1–3]. Quadrelli et al. reported that mortality is higher in patients who require dialysis and/or mechanical ventilation and SaO_2_ under 90% at admission [1]. Zamora et al. showed that the factors associated with increased mortality include mechanical ventilation, infection, and cyclophosphamide therapy [2]. Kohashi et al. proposed that factors for poor prognosis are a lactate dehydrogenase level over 230 IU/L, PaO_2_/FiO_2_ ratio under 300, and consolidation shadows on computed tomography [3]. The condition of our patient, with respiratory distress requiring mechanical ventilation support, active infective endocarditis, and consolidation shadows on images, was presumed to be very serious. Although surgical intervention was considered indispensable for the treatment of this endocarditis, we concluded that the patient would be unable to tolerate the surgery at first. We surmised that a procedure for respiratory functional improvement by excreting the clot from the alveolar space would be essential to obtain a successful surgical outcome in this situation. Although initially not sufficient, remarkable improvement in respiratory distress was achieved by aggressive respiratory rehabilitation with frequent positional change following the tracheotomy. The patient consequently underwent aortic valve replacement without any respiratory and infectious complications.

We adopted subannular reinforcement [5] using a woven Dacron graft in aortic valve replacement to prevent valve detachment. Although other surgeons consider the technique troublesome and time-consuming, this procedure might be an effective adjunct for pathology with a potentially fragile annulus.

In conclusion, we describe a patient who survived aortic valve replacement for bacterial endocarditis complicated by DAH. We believe that improving respiratory distress by aggressive respiratory rehabilitation to excrete any clots from alveoli is essential for successful cardiac surgery in patients with DAH.
